# Combined Diagnosis of Systemic Lupus Erythematosus and Tuberculosis in an Irish Adolescent Female

**DOI:** 10.1155/2018/2031219

**Published:** 2018-03-04

**Authors:** Kene Ebuka Maduemem, Comfort O. Adedokun, Adela Vatca

**Affiliations:** ^1^Department of Paediatrics, Cork University Hospital, Cork, Ireland; ^2^Department of Emergency Medicine, Cork University Hospital, Cork, Ireland

## Abstract

Systemic lupus erythematosus (SLE) is an autoimmune disease of unknown aetiology, which can affect any organ system. Tuberculosis (TB) is a common infection in SLE because of immune dysregulation associated with the latter. We report a case of an adolescent female who presented with a year's history of polyarticular arthralgia and fever. Physical examination revealed a large left effusion that needed drainage. Investigations revealed a combined diagnosis of SLE and TB. Management comprised quadruple anti-TB therapy and SLE treatment. She made a steady recovery and has maintained a stable state from the lupus perspective.

## 1. Introduction

Systemic lupus erythematosus (SLE) is a chronic multisystem autoimmune disease characterized by its protean manifestations. Infections contribute to the burden of morbidity and mortality in SLE [1]. The impaired cellular and humoral immune functions seen in SLE patients are predisposing conditions. Tuberculosis (TB) is a common infection among patients with SLE, especially in endemic countries. The main reasons for the high incidence of TB infection are immunosuppressive therapy and immune disturbances of lupus itself. TB infection and other opportunistic infections account partly for the increased mortality in SLE. Several studies have shown an increased predisposition to TB infection among SLE patients especially in TB endemic countries. Our reported case is presented to highlight the consequence of immune dysregulation in SLE on TB infection risk in a nonendemic country.

## 2. Case Report

A 14-year-old girl presented with 5-day history of continuous high-grade pyrexia on a background of a year's history of mild intermittent polyarticular arthralgia. The joints involved were the wrists, fingers, elbows, knees, ankles, and toes. There was no associated swelling, redness, or deformity. The joint pains were not associated with early morning stiffness. Her immunizations were up-to-date including BCG. She is the only child of Irish parents. Review of system revealed nondistressing chronic cough, anorexia, and occasional night sweats. History of TB contact was unknown. She had occasional malar rash which was thought to be flushing of cheeks.

Examination findings revealed a febrile girl in mild respiratory distress. She appeared pale, no digital clubbing or peripheral lymphadenopathy. Her vital signs were as follows: temperature 38.50°C, pulse rate 115 beats/minute, respiratory rate 32/minute, blood pressure 100/58 mmHg, and oxygen saturation 88% in room air. There was decreased chest expansion on the right. Air entry was diminished on the right upper and mid zones but absent at the base. Stony dull percussion on the right hemithorax was elicited.

**Figure 1:**
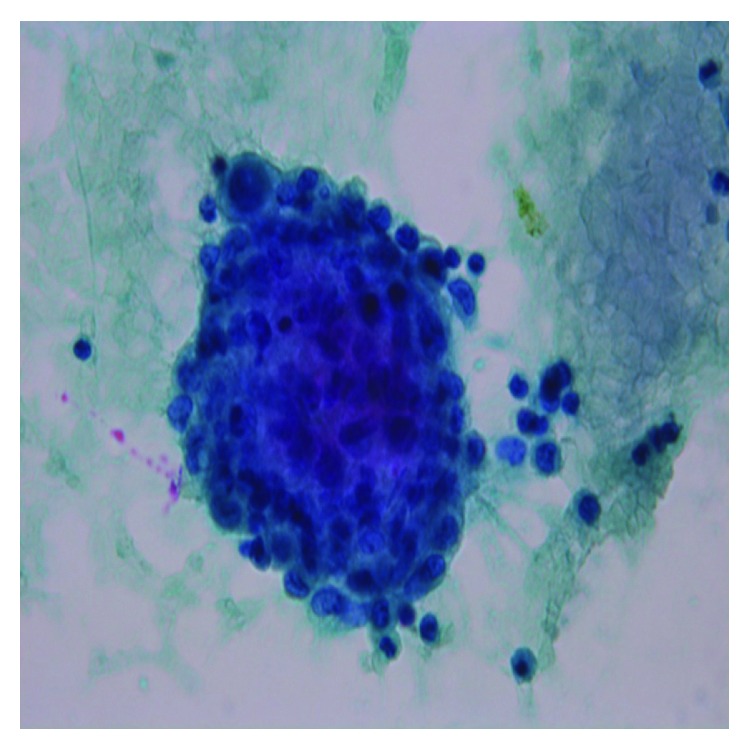
Pleural fluid cytology, PAP stain (×40): mesothelial cells with moderate number of lymphocytes admixed with macrophages and polymorphonuclear cells. Occasional loose aggregates of histiocytes.

**Figure 2:**
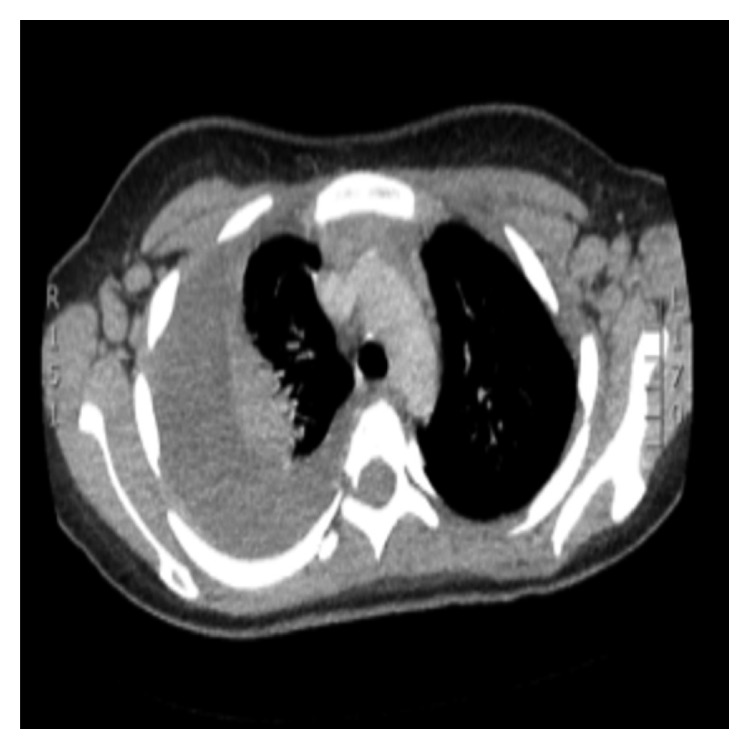
Contrast-enhanced CT thorax: large loculated right-sided pleural effusion. Small left-sided effusion. Bilateral axillary lymphadenopathy. Bulky left hilum.

**Figure 3:**
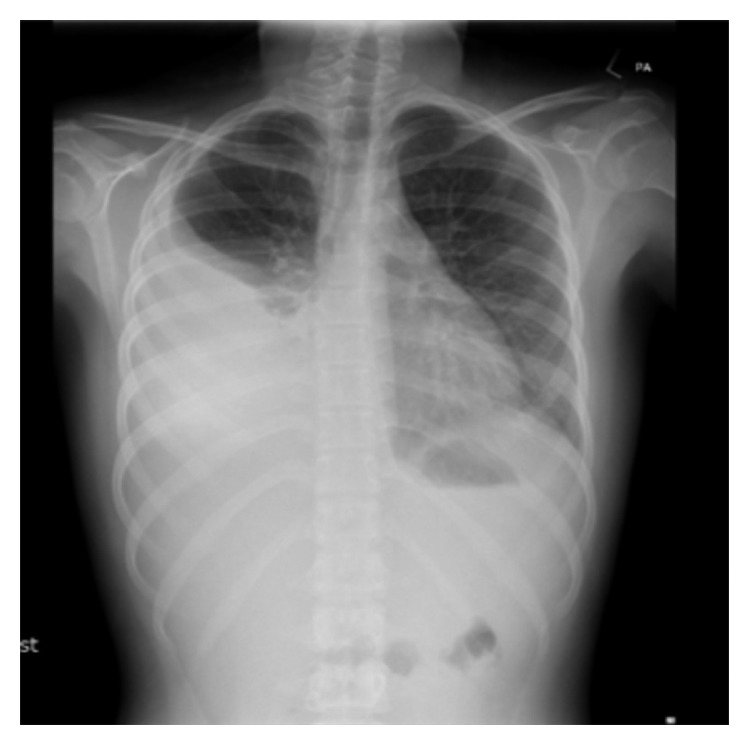
PA erect chest radiograph: large volume right-sided pleural effusion with atelectasis of right mid and lower lobes.

Full blood count showed haemoglobin level 11 g/dL, white cell count 4000/mm^3^ (neutrophils 60%, lymphocytes 31%, and monocytes 9%), and platelet count 243,000/mm^3^. MCV was 71.7 fL, and MCH was 24 pg. Erythrocyte sedimentation rate (ESR) was 83 mm/hour, and C-reactive protein (CRP) was 54.8 mg/L. Lactate dehydrogenase (LDH) was 465 U/L, and urate was 401 *µ*mol/L. The renal function and liver function tests were normal. Autoimmune screening showed that anti-nuclear antibody (ANA) was strong positive homogenous and anti-double-stranded DNA was >379 IU/ml. Other positive antibodies include extractable nuclear antibody, crithidia anti-double-stranded antibody, and anti-Ro and anti-La antibodies. IgM rheumatoid factor was <11.4 IU/mL. Serum ACE level was 47 U/L, and low C3 and C4 levels were at 0.73 and <0.08 g/L, respectively. Quantiferon TB1 and TB2 were 2.34  and 1.75 IU/ml, respectively. Pleural fluid biochemical analysis showed pH 7.25, protein 50 g/L, glucose 64 mg/dL, and LDH 256 U/L. There was pleural fluid lymphocytosis (85%) with no culture growth. Pleural fluid cytology showed loose aggregates of histiocytes and poorly formed granuloma (Figure 1). Bronchial lavage sample yielded no growth on culture. Chest radiography (Figure 2) demonstrated moderate to large volume right-sided pleural effusion. Contrast-enhanced computed tomography (CT) scan of thorax showed large right pleural effusion, bilateral axillary lymphadenopathy, and a bulky left hilum (Figure 3). The Mantoux test (before immunosuppressant therapy) was negative.

She was initially treated as a case of parapneumonic effusion. She received a 4-day course of antibiotics. Ultrasound-guided pigtail drain was inserted on her right chest, which drained approximately 2000 ml of serosanguinous fluid over 24 hours. Rheumatology team in view of SLE commenced her on a 3-day course of intravenous methylprednisolone and a 10-day course of tazobactam/piperacillin. She was continued on high-dose oral prednisolone. Other medications include hydroxychloroquine 200 mg once daily, mycotil mycophenolate (CellCept) 250 mg 12 hourly, and omeprazole 20 mg once daily. Following review of investigations by infectious disease team, she was started on quadruple anti-TB treatment (isoniazid, rifampicin, pyrazinamide, and ethambutol) and pyridoxine.

There was gradual but steady clinical improvement while on the SLE and TB treatments. Chest radiograph at 15 weeks of treatment showed near-complete resolution of effusion. She was on regular follow-up with the dietician, occupational therapist, and physiotherapist. Her inflammatory markers have returned to normal limits. She is currently enjoying school having been on treatment for 5 months.

## 3. Discussion

TB has a higher propensity under immunosuppressive states and may be either secondary to the disease or because of immunosuppressive therapy. TB is an important public health problem globally. TB, in 2016, was listed as one of the ten causes of deaths globally [2]. In the European Union (EU), it unfortunately remains an unresolved issue. This may be accounted for by high rates of immigrants from TB endemic countries.

Our patient presented with varied signs and symptoms which were nonspecific initially. She was subsequently thought to have SLE owing to the autoimmune panel. SLE could tie up all her symptoms ranging from arthritis to serositis. However, the QuantiFERON-TB Gold test was requested despite no documented history of TB contact or foreign travel. It was quite unusual to have a diagnosis of latent TB infection (LTBI) versus TB disease in a fully vaccinated Irish resident without documented contact or travel. The two disease processes often mimic each other, making the diagnosis more challenging.

Several theories have been postulated to explain the increased propensity of SLE patients to develop TB. One theory is that high doses of corticosteroids and/or other immunosuppressive agents are main causes [3, 4]. However, other reports suggest that the disease itself might contribute to the increased risk [5]. Studies have documented increased susceptibility for TB among SLE patients; however, these revolved around endemic countries [6, 7]. Patients with SLE are prone to infections owing to abnormality in their immune system: immunoglobulin deficiency, complement deficiencies, defects in chemotaxis, phagocytosis, delayed hypersensitivity, and abnormalities of cellular immunity [8]. Erdozain et al. reported a 6-fold higher incidence of TB in the SLE group as compared to the general population [9]. Similarly, Mok et al. reported a 5- to 15-fold higher risk [10]. Cellular immune responses are involved in the control of *Mycobacterium tuberculosis* infection. The impaired cellular and humoral immune functions in SLE patients have been demonstrated to be associated with developing clinically manifested TB.

The tuberculin skin test (TST) can be useful in the diagnosis of active TB. However, the TST has been reported to be significantly anergic in patients with SLE [11]. The TST is limited by low sensitivity in immunocompromised patients and low specificity due to cross reactivity with other nontuberculous mycobacteria. The QuantiFERON-TB Gold test is an interferon-gamma release assay (IGRA) that measures the release of interferon-gamma after in vitro stimulation by *Mycobacterium tuberculosis* antigens using ELISA [12]. IGRAs are rarely influenced by prior BCG vaccinations and, thus, superior to TST for immunocompromised conditions [3]. Serial IGRA measurements can be a valuable tool in detecting noncompliance of medications in active TB treatment or prophylaxis [13]. It has been shown that IGRAs have a higher specificity for LTBI, especially in low-TB-burden settings, BCG-vaccinated children, and those exposed to nontuberculous mycobacteria [14]. Although our patient may have TB based on positive IGRA, since this test is not specific for active TB and the findings of negative cultures, it is highly possible that the clinical and laboratory findings are solely due to SLE. Despite ongoing steps to possibly divert from universal to selective BCG vaccination in the Republic of Ireland [15], the authors argue that, given the rate of immigration, all children will potentially benefit from BCG vaccination.

## 4. Conclusion

To the best of our knowledge, this is the first reported case of SLE and TB coinfection in the paediatric age group in the Republic of Ireland. Surveillance of TB infection in paediatric SLE is warranted regardless of TB endemicity. The authors highlight that sustaining BCG vaccination programme in the Republic of Ireland might be worthwhile as the battle against TB infection has not entirely been won.

## Abbreviations

BCG: Bacillus Calmette-Guerin
ELISA: Enzyme-linked immunosorbent assay
LDH: Lactate dehydrogenase
SLE: Systemic lupus erythematosus
TB: Tuberculosis.
